# Mining the Australian Grains Gene Bank for Rust Resistance in Barley

**DOI:** 10.3390/ijms241310860

**Published:** 2023-06-29

**Authors:** Md Arifuzzaman, Matthias Jost, Meinan Wang, Xianming Chen, Dragan Perovic, Robert F. Park, Matthew Rouse, Kerrie Forrest, Matthew Hayden, Ghazanfar Abbas Khan, Peter M. Dracatos

**Affiliations:** 1 Department of Genetics and Plant Breeding, Hajee Mohammad Danesh Science and Technology University, Dinajpur 5200, Bangladesh; 2 CSIRO Agriculture and Food, Commonwealth Scientific and Industrial Research Organisation, Canberra, ACT 2601, Australia; 3 Department of Plant Pathology, Washington State University, Pullman, WA 99164-6430, USA; 4 Agricultural Research Service, United States Department of Agriculture Wheat Health, Genetics and Quality Research Unit, Pullman, WA 99164-6430, USA; 5 Federal Research Centre for Cultivated Plants, Institute for Resistance Research and Stress Tolerance, Julius Kuehn-Institute, Erwin-Baur-Strasse 27, 06484 Quedlinburg, Germany; 6 Plant Breeding Institute, Faculty of Science, The University of Sydney, Cobbitty, NSW 2570, Australia; 7 USDA-ARS Cereal Disease Laboratory and Department of Plant Pathology, University of Minnesota, St. Paul, MN 55108, USA; 8 Agriculture Victoria Research, AgriBio, Melbourne, VIC 3083, Australia; 9 Department of Animal, Plant and Soil Sciences, La Trobe University, AgriBio, Bundoora, VIC 3086, Australia

**Keywords:** genotyping-by-sequencing (GBS), barley rust diseases, genome-wide association study (GWAS)

## Abstract

Global barley production is threatened by plant pathogens, especially the rusts. In this study we used a targeted genotype-by-sequencing (GBS) assisted GWAS approach to identify rust resistance alleles in a collection of 287 genetically distinct diverse barley landraces and historical cultivars available in the Australian Grains Genebank (AGG) and originally sourced from Eastern Europe. The accessions were challenged with seven US-derived cereal rust pathogen races including *Puccinia hordei* (*Ph*-leaf rust) race 17VA12C, *P. coronata* var. *hordei* (*Pch*-crown rust) race 91NE9305 and five pathogenically diverse races of *P. striiformis* f. sp. *hordei* (*Psh*-stripe rust) (PSH-33, PSH-48, PSH-54, PSH-72 and PSH-100) and phenotyped quantitatively at the seedling stage. Novel resistance factors were identified on chromosomes 1H, 2H, 4H and 5H in response to *Pch*, whereas a race-specific QTL on 7HS was identified that was effective only to *Psh* isolates PSH-72 and PSH-100. A major effect QTL on chromosome 5HL conferred resistance to all *Psh* races including PSH-72, which is virulent on all 12 stripe rust differential tester lines. The same major effect QTL was also identified in response to leaf rust (17VA12C) suggesting this locus contains several pathogen specific rust resistance genes or the same gene is responsible for both leaf rust and stripe rust resistance. Twelve accessions were highly resistant to both leaf and stripe rust diseases and also carried the 5HL QTL. We subsequently surveyed the physical region at the 5HL locus for across the barley pan genome variation in the presence of known resistance gene candidates and identified a rich source of high confidence protein kinase and antifungal genes in the QTL region.

## 1. Introduction

Wholegrain cereals are a rich source of carbohydrates, proteins, vitamins, minerals and phytochemicals, providing both general nourishment and immunological benefits against diseases such as high cholesterol and cancer [1]. Barley is the world’s fourth most important cereal, used primarily in malt production for alcoholic beverages and as grain feed for livestock and human food. As the effects of climate change become more prevalent, the importance of barley is likely to increase due to its ability to yield in marginal environments. Of concern, though, are foliar diseases that reduce yield, grain quality and profitability [2]. Barley is affected by four distinct rust diseases: stripe rust (*Puccinia striiformis* f. sp. *hordei*; *Psh*), leaf rust (*P. hordei*; *Ph*), stem rust (*P. graminis* f. sp. *tritici*; *Pgt*) and crown rust (*P. coronata* var. *hordei*; *Pch*). Whilst resistance to leaf rust [3] and stem rust [4,5] is widely available and well characterised for barley, fewer stripe rust resistance loci have been formally characterized [6,7,8], and resistance to crown rust in barley is poorly understood, with only one mapped resistance locus *HvPc1* on chromosome 3H [9].

Cereal breeding has historically been influenced by changing political constraints and the gene flow of germplasm, largely mirroring human movement over time. Mining germplasm collections or gene pools for traits of interest and introducing them into crop breeding programs is far from novel but is an effective approach to diversify disease resistance alleles in breeding systems. Fundamental to this activity are the tens of thousands of crop accessions maintained in global gene banks along with passport data (a basic description of the accession such as accession name, genus, country of origin, acquisition date, etc.). Due to the historical movement of cereal germplasm within and between Western countries (Europe, USA, UK and Australia) it is hypothesized that the barley gene pool from Eastern Europe has been largely under-utilised for disease resistance breeding.

Resistance breeding is often constrained by the narrow genetic base in modern adapted crop varieties and the lack of allelic diversity present in traditional biparental crosses. The genome-wide association study (GWAS) approach overcomes several limitations of traditional gene mapping by (i) providing higher genetic mapping resolution by exploiting historical recombination in populations with appropriate linkage disequilibrium and (ii) enabling the mining of diverse germplasm collections for rare allelic variants associated with phenotypic variation. Previously developed advancements in genomic technologies such as genotyping-by-sequencing (GBS) now permit increased marker numbers and affords enhanced precision and power in quantitative genetic approaches such as GWAS and genomic selection [10]. In this study we assembled a diverse collection of 318 barley accessions from the Australian Grains Genebank (AGG) that were originally sourced from countries spanning Eastern Europe (“Eastern European Barleys”; EEBs). GBS analysis enabled the identification of >30 K single nucleotide polymorphism (SNP) markers and further resolved the 318 EEBs to 287 non-redundant genetically distinct accessions. Using GWAS we identified several genomic regions contributing novel resistance to barley infecting cereal rust pathogen isolates that are potentially suitable for introgression into modern high-yielding barley cultivars.

## 2. Results

### 2.1. Population Structure

**Figure 1:**
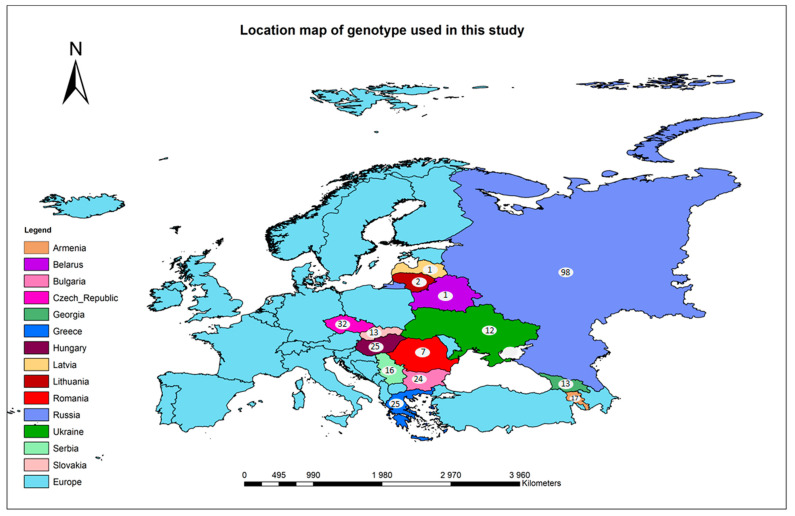
Geographic distribution of 287 Eastern European Barley accessions used in the study.

**Figure 2:**
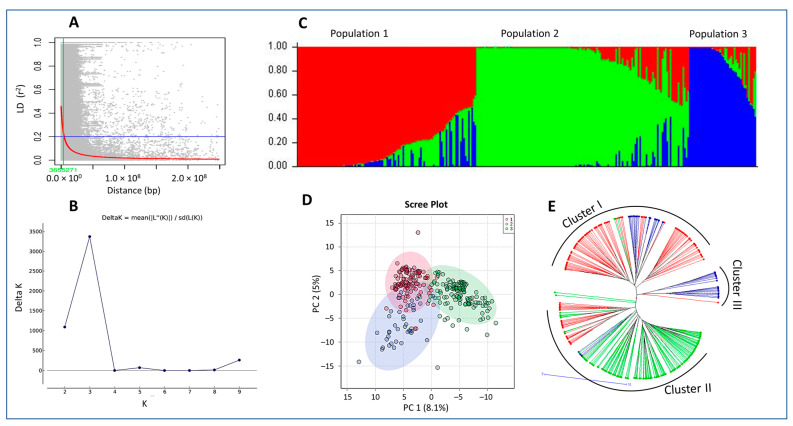
Linkage disequilibrium (LD) and population structure analyses of the 287 Eastern European Barley (EEBs) accessions (**A**) Genome-wide average LD decay over physical distance. Pair-wise single-nucleotide polyLD (r^2^) values based on the physical positions from the Morex reference genome assembly (v1) [11] were plotted as a function of mapping distance (bp) between markers. The red colour curve represents the LD decay across the whole genome. The thick horizontal blue line represents the population-specific critical r^2^ value (0.2) above which LD may be due to linkage, (**B**) Population structure of a panel of 287 genetically distinct EEB accessions based on 1073 molecular markers (K = 3), (**C**) Admixture model of structure of *ΔK* for EEB populations, (**D**) Two-dimensional PCA biplot, (**E**) A neighbour-joining (NJ) phylogenetic tree of the 287 barley accessions, here, red, green and blue colours indicate the barley genotypes derived from Population I, 2 and 3, respectively.

The EEB accessions were sourced based on origin from 16 different counties in Eastern Europe (Figure 1; Supplementary Table S1). Most accessions (98) were collected from Russia or the former Soviet Union, followed by Czech Republic (32), Greece (25) and Hungary (25). A total of 31,805 SNP markers that were polymorphic across the entire EEB panel was used to identify duplicated accessions, which resulted in the identification of 287 genetically distinct non-redundant EEBs. An LD decay (LD ≤ 0.2) based on and average distance of 1 SNP every ~3,655,271 bp was the basis of selecting 1073 SNP markers for population structure analysis (Figure 2A). Population structure analysis performed on the non-redundant EEBs using 1073 SNPs selected from the LD decay analysis estimated the membership fractions to range 2 to 10 (Figure 2B). Structure analysis showed the 287 accessions could be clustered into three distinct populations based on the most significant Evanno’s Δ*k* of 3 [population 1 (39.02%), population 2 (46.35%) and population 3 (14.63%)]. Membership fractions were used to classify the populations as either pure or admixed genotypes, which revealed that >65% of the total members for each population were pure [population 1 (65.18%), population 2 (75.19%) and population 3 (69.05%)] (Figure 2C).

Principal Component Analysis (PCA) using genotypic data for the 287 non-redundant EEBs showed that PC1 and PC2 explained 8.1% and 5% of the total variation, respectively, as depicted in the 2D PCA plot in Figure 2D. The clustering for the kinship matrix was broadly correlated with the clustering in the NJ phylogenetic tree despite a degree of admixture suggesting that the 287 EEB accessions clustered into three sub-populations, corroborating the Structure analysis (Figure 2E). Genetic distance among the populations was calculated, and the largest distance was between populations 2 and 3 (0.2) followed by populations 1 and 2 (0.19). The fixation index F_ST_ was used to measure the population differentiation among the 287 EEBs due to genetic structure. As expected, greater differences were observed between the three populations relative to the diversity of individuals within populations [F_ST_ population values: population 1 (0.42), population 2 (0.58) and population 3 (0.44)] with an average of alpha 0.11 representing the significant differences among the population structure. Average distances (expected heterozygosity) between individuals within the same population were calculated and the average genetic distance (net nucleotide distance) between populations 1 and 2 was 0.19, populations 2 and 3 was 0.2 and populations 1 and 3 was 0.14.

### 2.2. Phenotypic Rust Response under Controlled Greenhouse Conditions

**Figure 3:**
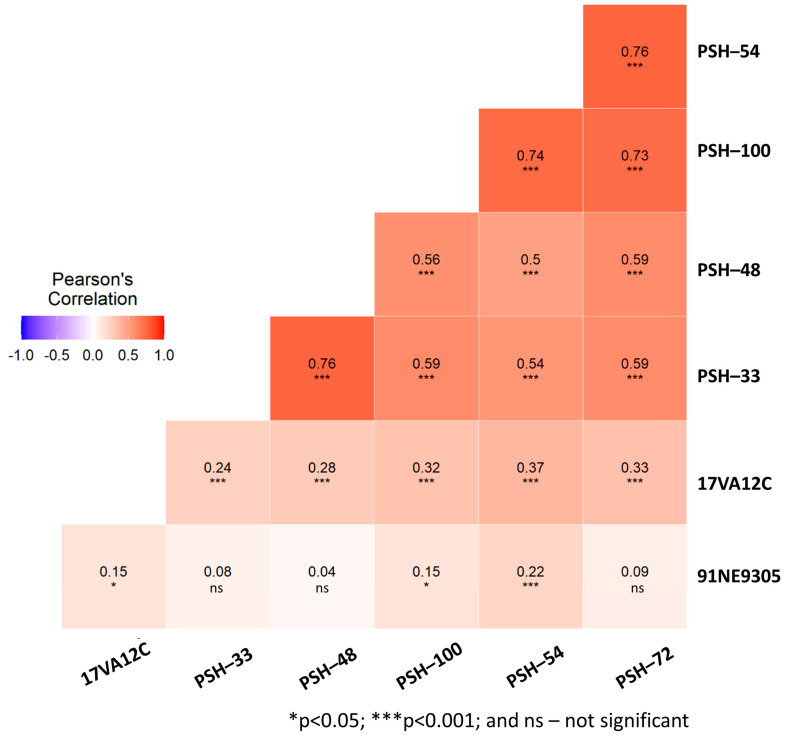
Pearson’s correlation coefficients comparing the phenotypic pair-wise correlations between seven rust traits assessed in the Eastern European Barley accessions. Significance for the Pearson’s Correlation was assessed at *p* < 0.05 (*) and *p* < 0.001 (***).

The 287 EEB lines were assessed phenotypically at the seedling stage using a 1–9 scale in independent experiments with five pathogenically diverse *Psh* races (PSH-33, PSH-48, PSH-54, PSH-72 and PSH-100) at USDA-ARS and Washington State University (WSU) in Pullman, Washington and one race each of *Ph* (17VA12C) and *Pch* (91NE9305) at the Cereal Disease Laboratory of USDA-ARS at St. Paul, Minnesota. The phenotypic distribution frequencies for all nine exotic rust traits are shown in (Supplementary Figure S1). Most traits skewed towards susceptibility and in general revealed the presence of a limited number of rust resistant accessions. The virulence/avirulence spectra of each of the *Psh* races was assessed using 12 well-characterized barley stripe rust differential stock genotypes (Table 1). The phenotypic response results revealed that increased susceptibility in the EEBs to the *Psh* races tested was correlated with increased virulence on the 12 barley stripe rust differentials. For example, 88% of the EEBs were susceptible to the most virulent race PSH-72 (mean IT 7.9), whereas 55% were susceptible to avirulent race PSH-48 (mean IT 6.4), likely suggesting that resistance genes present in the differential lines were also present in specific EEB accessions. Pearson’s correlation coefficient data determined that the phenotypic response datasets for avirulent races PSH-33 and PSH-48 differing only for virulence on Abed Binder 12 were highly correlated (0.76) (Figure 3). These avirulent *Psh* races were less correlated (0.5–0.6) with the phenotypic response data of the other three more virulent races (PSH-54, PSH-100 and PSH-72) which were highly correlated with each other (0.73–0.76) (Figure 3). Four lines were highly resistant (HR 0–3), five moderately resistant (MR 4–6) and 16 lines were either MR or HR to all races, suggesting the presence of both broadly effective and race-specific resistance components. EEB accessions from Greece, Russia, the Czech Republic and the Former Soviet Union were the most resistant to PSH-72, suggesting that they contained largely uncharacterized resistance given that this pathogen race was virulent on all the genes in the differential set.

Phenotypic evaluations of the 287 EEB accessions to other rusts were performed at the Cereal Disease Laboratory, St. Paul, MN, USA. Replicated infection type (IT scale 0–4) response data were recorded at the seedling stage using the barley crown rust (*P. coronata* var *hordei*- race 91NE9305) and barley leaf rust (*P. hordei* race 17VA12C) pathogen isolates. All EEB lines assessed were either susceptible (88%) or moderately susceptible (10%) to barley crown rust race 91NE9305. Only six lines (2%) were highly resistant, accounting for the poor correlation (0.04–0.15) between 91NE9305 and other rust traits with the exception of moderate correlation (0.22) with PSH-54 (Figure 3). In contrast, unexpectedly, resistance to *P. hordei* was significantly correlated (*p* < 0.001) with all five PSH races relative to crown rust. Sixteen EEB accessions, mainly from the Czech Republic/Slovakia (7), Russia/Soviet Union (6) and Greece (3) were resistant to leaf and either most or all stripe rust isolates used in this study, suggesting a possible correlation in the underlying genetic control of these traits.

### 2.3. Marker–Trait Associations

**Figure 4:**
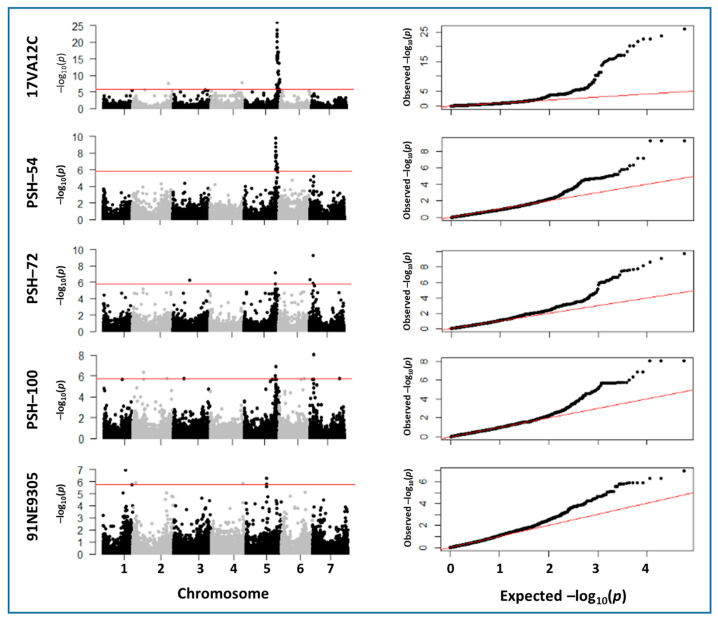
Manhattan plots of the SNPs (*n* = 28,780). The horizontal lines indicate the threshold value at −log10(*p*-value) = 5.76. Plots displayed across the seven barley chromosomes indicate the SNPs associated with resistance to five out of the seven rust traits assessed on the Eastern European Barley accessions. Quantile–quantile plots are displayed on the right of each Manhattan plot.

A total of 14 significant marker–trait associations (MTAs) were detected [−log10 (*p*-value) > 5.8], which represented nine different QTL regions for five rust resistance traits across all seven chromosomes (with the exception of 6H) as displayed using Manhattan plots and Quantile–Quantile (Q-Q) plots (Table 2; Figure 4). For barley stripe rust, significant MTAs were identified for three (PSH-54, PSH-72 and PSH-100) out of five PSH races tested. The same highly significant QTL on chromosome 5HL was identified and effective to all three *Psh* isolates, whereas a second QTL on chromosome 7HS was significant in response PSH-72 and PSH-100 but under the significance threshold for PSH-54. Race-specific singleton QTLs were identified on chromosomes 2H and 3H in response to race PSH-100 but not PSH-54.

**Figure 5:**
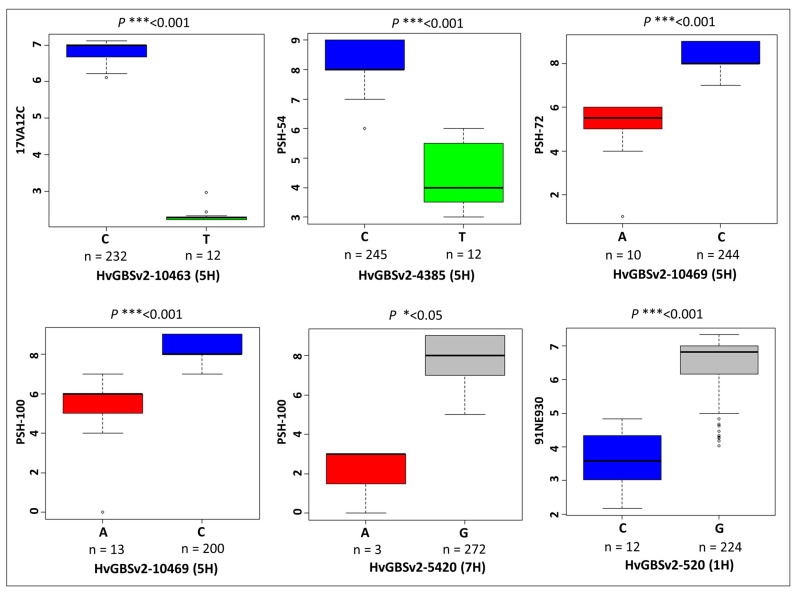
Boxplots showing the median phenotype of Eastern European lines for the most significant SNP marker allele associated with either resistance or susceptibility to the respective rust traits that were identified using GWAS. Statistical significance was measured using a two-tailed *t*-test where statistical significance is denoted as *p* < 0.05 (*) and *p* < 0.001 (***).

Three QTL were identified in response to leaf rust race 17VA12C. The peak marker for the most significant QTL identified in response to 17VA12C [−log_10_(*p*-value) = 25.95] and was located on chromosome 5HL within a 5 Mb distance to the peak markers identified for broadly effective 5HL QTL for resistance to *Psh* races PSH-54, PSH-72 and PSH-100 (Table 2). Of the remaining QTL, two were identified on chromosome 3H and the other on chromosomes 4HS, 5HS and 6HL. Two other minor effect QTL were also identified on the long arms of chromosome 2H and 4H respectively. Two clearly significant resistance QTL were identified in response to *Pch* race 91NE9305 on the long arms of chromosomes 1H and 5H, in addition to two minor effect QTLs identified on 2HS and 4HL. Interestingly, the same peak marker was identified for the 4HL QTL in response to both *Ph* and *Pch* races, suggesting that the same gene may contribute resistance to both rust diseases. To further validate the main QTLs identified in this study we created box plots and performed a two-tailed *t*-test to observe the effect of alternative SNP marker alleles on rust phenotypic response. For the major 5HL QTL we identified a highly significant effect (*p* < 0.001) between alternative marker alleles on the mean phenotypic response to *Ph* race 17VA12C and all three *Psh* races (PSH-54, PSH-72 and PSH-100) (Figure 5).

### 2.4. Investigating Genomic Regions Carrying Markers with Association to Multiple Pathogens

**Figure 6:**
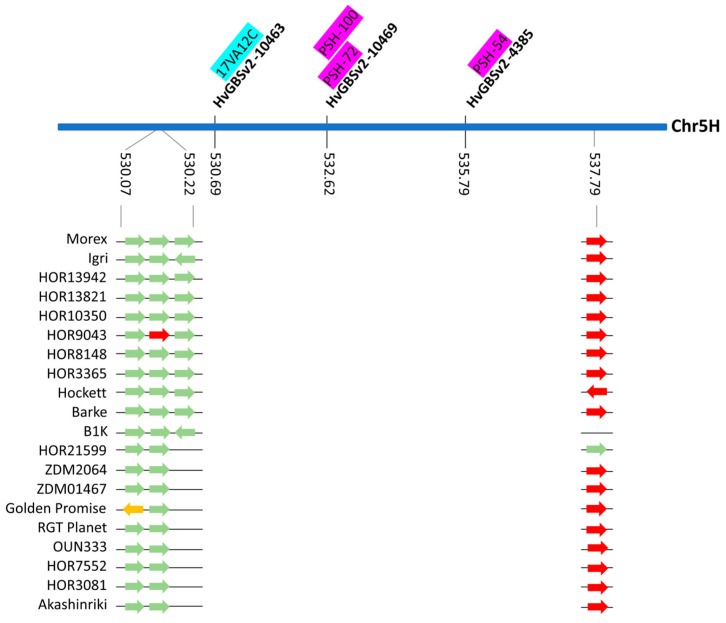
Schematical drawing of markers associated with leaf rust (cyan), stripe rust (purple) and 5H. Genomic coordinates for markers and detected NLR genes are given based on MorexV3 reference genome [12]. Predicted NLRs are based on automatic motif search using the NLR annotator [14] for the 20 accessions of the barley pan-genome are illustrated as directional arrows, where complete NLRs are highlighted in green, complete pseudogenes as orange and partial NLRs as red arrows.

A locus carrying SNPs associated with more than one pathogen was identified in close proximity. One marker associated with *Ph* isolate 17VA12C and two markers associated with resistance to *Psh* isolates PSH-100, PSH-72 or PSH-54, respectively, were detected within 5 Mb on chromosome 5H (Figure 6, Table 2). The close proximity of race-specific genes could indicate an interesting resistance gene cluster on the long arm of chromosome 5H. To investigate this region further, sequence for the corresponding regions including 5Mb of flanking sequence was extracted from the Morex v3 [12]) reference genome assembly and corresponding regions in the 19 accessions of the barley pan-genome [13]. Initial analysis of the sequence showed that marker order, sequence length and overall gene content between the 20 accessions was comparable (Supplemental Figure S2). We firstly focused on annotating members of the major category of race-specific resistance genes by performing a sequence motif search for NBS-LRR resistance for the respective regions in the 20 barley genomes. A single NLR about 2 Mbp upstream of the PSH-54 associated marker HvGBSv2-4385 was detected in most of the accessions. A cluster of NLRs was identified 470 kb downstream of marker HvGBSv2-10463 associated with resistance to *Ph* (Figure 6). The results indicate that multi-pathogen resistance is likely controlled by separate individual resistance genes.

## 3. Materials and Methods

### 3.1. Plant Materials

To identify resistance at the seedling stage, 318 diverse barley accessions were initially selected based on their Eastern Europe origin, and the seeds were provided by the Australian Grains Genebank (AGG, Horsham, VIC, Australia). In summary, the Eastern European Barley Collection (EEB *n* = 318) was further assessed for genetic redundancy (summarized in later section) resulting in 287 genetically unique accessions comprising both cultivated barley accessions, including landraces, historical cultivars, breeding lines and 145 with unknown origin (Refer to Supplemental Table S1 for passport data). Purification of each of the EEB accession sourced from the AGG was performed by growing only one seed from each purified seed increase. Seed derived in this way was used for rust testing and DNA extraction.

### 3.2. Pathogen Isolates

The panel was assessed with seven isolates of several barley infecting cereal rust pathogens from the USA. Five US races of the barley stripe rust pathogen *P. striiformis* f. sp. *hordei* (PSH-33, PSH-48, PSH-54, PSH-72 and PSH-100) were selected for phenotypic assessment of the EEB accessions. These *Psh* races were selected due to their prevalence in the USA and their wide virulence spectra across the 12 barley stripe rust differential tester genotypes (Table 1). The inoculum of all *Psh* isolates used in this study was increased on seedling susceptible barley lines Steptoe or Topper. The EEB accessions were also assessed with single-pustule-derived races of *P. coronata* var *hordei* (91NE9305) and *P. hordei* [(17VA12C) with virulence/avirulence formulae (*Rph1*, *Rph2*, *Rph4*, *Rph5*, *Rph6*, *Rph7*, *Rph9*, *Rph10*, *Rph11*, *Rph13*, *Rph19*/*Rph3*, *Rph12*, *Rph14*, *Rph15*)], which were provided by Dr Matthew Rouse (USDA-ARS) with the aim of identifying and mapping resistance to each of these barley rust pathogen isolates.

### 3.3. Inoculation and Phenotypic Assessment of Rust Resistance

For the assessment of resistance to *Psh* in the greenhouse at the seedling stage, 5–7 seedlings per plant genotype were inoculated for each race test and scored on an ordinal scale of 1–9 as previously described [7,8]. A uniform dry inoculation was applied to seedlings at the two-leaf stage using a mixture of fresh urediniospores and talc [15]. Once inoculated, the seedlings were placed in a dew chamber (18–24 h) followed by a temperature-controlled greenhouse on a diurnal cycle. Infection on the EEBs and the 12 *Psh* differential barley lines were assessed phenotypically 18 days following inoculation. For resistance to *Psh*, three phenotypic classes were used to classify the EEB tester lines including highly resistant (0–3), moderately resistant (4–6) and susceptible (7–9).

The barley leaf rust differentials [3] were included as controls for both the leaf and crown rust experiments. Seedling infection types were scored on a 0–4 scale as described in [3] for barley leaf and crown rust. Assays for barley seedling response to *P. coronata* var *hordei* were performed like those for *P. hordei* except plants were maintained at 20–22 °C after inoculation through disease assessment [5,16]. The seedling infection type data were linearized to a 0–9 scale according to Gao et al. [17], however the linearized data were not transformed. The data from the two replications of each race were averaged. Barley leaf rust and barley crown rust were scored 10–11 days after inoculation. Two replications of the EEB panel were evaluated for response to each rust pathogen isolate in separate experiments with no randomisation.

### 3.4. Genomic DNA Isolation and Quantification

A healthy portion of fresh youngest leaves from two-week old plants from each of the 318 EEB accessions were collected and desiccated for 5–7 days. DNA extraction was performed following the modified CTAB method described by Fulton et al. [18]. The DNA pellets obtained using this protocol were allowed to dissolve in TE buffer and were stored at 4 °C in a refrigerator. The following day, RNase A treatment was performed (10 µg/mL) followed by incubation at 37 °C for 30 min. The quality of the extracted DNA samples was checked using an 0.8% agarose gel using and quantified using a Thermo ScientificNanoDrop^TM1000^ Spectrophotometer (Thermo Fisher Scientific, Lenexa, KS, USA).

### 3.5. Genotyping of Barley Genotypes

A targeted GBS (tGBS) assay designed against 11,851 loci evenly distributed across the barley genome was deployed by Agriculture Victoria was used to genotype the 318 accessions in the EEB panel, to perform population structure analysis and GWAS for rust resistance traits at the seedling stage. Sample reads from the tGBS assay were used to generate genotype calls for all polymorphic loci. First, the sample read data are used to build an allele-specific reference. Next, allelism among the allele-specific reference sequences was determined from their alignment to the Morex reference genome assembly v1 [11]. Variant genotype calls were reported as homozygous ‘0′ or ‘2′, or heterozygous ‘1′, where ‘0′ referred to the REF variant and 2 referred to the ALT variant. Missing data were represented by ‘U’.

### 3.6. Identifying Genetically Redundant Accessions

Variant calling with tGBS data of 318 barley genotypes was carried out essentially as described by Milner et al. [9]. Reads were trimmed with cutadapt [19] and mapped to the MorexV3 reference genome sequence assembly [12] with BWA-MEM [20]. Mapping records were converted to Binary Sequence Alignment/Map (BAM) format with Samtools [21] and sorted with Novosort (http://www.novocraft.com/products/novosort/ (accessed on 30 April 2022). Variant calling was carried out with bcftools [22]. A custom script was used for variant filtration (https://bitbucket.org/ipk_dg_public/vcf_filtering (accessed on 30 April 2022). The final variant matrix contained bi-allelic SNPs with less than 10% missing data and less than 1% heterozygous calls per site. An identity-by-state (IBS) matrix was calculated with PLINK [23] and imported into the R statistical environment [24] (R Core Team 2022). Clusters of duplicated accessions were found by identifying connected components in the graph induced by the IBS matrix using igraph [25]. Two accessions were considered duplicates if they had present genotypes that call at least 1000 common SNP sites, and these genotype calls were identical in at least 99.8% of cases.

### 3.7. Imputation

For input into imputation, the genotypic data for the SNP/INDEL calls meeting were set as samples with a 25% call rate, markers with a 40% call rate and 1% minor allele frequency (MAF). Eight samples were excluded from imputation due to call rate < 25%. Missing marker data were imputed using LinkImpute [26], which imputes missing genotype data with 90% accuracy based on a k-nearest neighbor genotype imputation method that is designed for unordered markers. A total of 31,805 markers were selected for further analysis.

### 3.8. Population Structure Analysis

Population structure was accounted for using STRUCTURE and Principal Component Analysis (PCA). From 31,805 SNP markers, a subset of 1073 SNP markers were selected based on LD decay analysis and used for population structure analysis. The software STRUCTURE v2.3.4 [27] was used to estimate the population structure of the non-redundant EEB accessions (*n* = 287) to create a population structure matrix (*Q*) to be used as a covariate. To determine the optimal number of sub-populations, an admixture ancestry model was used with a burn-in of 1,000,000 followed by 1,000,000 Monte Carlo Markov Chain (MCMC) replications for *k* = 1 to *k* = 10 with five iterations. STRUCTURE HARVESTER [28] was used to identify the optimal number of sub-populations using the Δ*k* method [29]. An individual was deemed to be part of a population if the membership probability was >0.8 [30]. Individuals that did not achieve a value of 0.8 were deemed to have admixture ancestry.

### 3.9. Linkage Disequilibrium

The SNPs linkage disequilibrium (LD) estimates were determined for pairs of loci using the software package Tassel 5.0 [31] using SNPs of known marker positions only. A squared allele-frequency correlation (r^2^) [32] was calculated for each intra chromosomal combination. The distribution and extent of LD were visualized by plotting intra-chromosomal r^2^ values against the genetic distance in cM for all inter-chromosomal marker pairs using nonlinear regression as described in [33] and implemented in R statistics of version 4.2.2 [24] (R Core Team 2022).

### 3.10. Detection of Significant SNP Markers Using GWAS

The SNP markers were further filtered using thresholds for minor allele frequency (MAF) of 0.01, missing value rate of 0.20 and heterozygosity of 0.20. The final filtered set of 28,780 SNPs was used for GWAS on the non-redundant EEB accessions (*n* = 287). There were 3427, 4495, 4957, 3293, 4897, 3798 and 3903 SNPs on chromosomes 1H to 7H, respectively. Based on the population structural analysis, a mixed linear model (MLM) was used to investigate best-fit models to search for SNP associations with the traits. The MLM model was selected as it considers population structure (Q) and relative kinship (K) effects and shows the best approximation of the expected cumulative distribution of *p*-values and is therefore more effective in controlling false positives. The population structure matrix (*Q* matrix) and the kinship matrix depicted using TASSEL 5.0 [31] was used for the model and GWAS analysis was performed for each trait using the phenotypic mean values. The standard Bonferroni-corrected threshold of α  =  0.05 was used as the significance cutoff. The suggested *p*-value was computed as 0.05/*n* (*n*  =  28,780), with the *p*-value of 1.70 × 10^−6^ [−log_10_(*p*-value) < 5.76] used as the final significance cut-off in the association analysis. Manhattan plots were constructed with the chromosome position on the X-axis against –log(*p*-value) of all SNPs, and quantile–quantile (QQ) plots of observed *p*-values were constructed against expected *p*-values using R Statistics of version 4.2.2 [24] (R Core Team 2022). The distribution of the QQ plot was considered to select the best model for each trait. The optimum model for each variable was determined as the one with the QQ plot with a smaller deviation from the normal distribution. The markers that significantly associated phenotypic traits were assigned to a QTL. The peak and flanking marker positions were converted from their bp position provided in Morex V1 [11] to Morex V3 [12].

### 3.11. Pan-Genomic Evaluation of Candidate Loci

Sequences surrounding SNP markers with the strongest associations (peak markers from Table 2) were used for BLAST to identify coordinates within the 20 accessions of the barley pan-genome [13]. The identified regions were extracted from pseudomolecules by using SAMtools version 1.12 [22] including a 5 MB downstream and upstream sequence. An NLR prediction and annotation was performed as previously described (https://github.com/steuernb/NLR-Annotator (accessed on 30 March 2023) using default parameters [14]. The gene descriptions were extracted from the gene projection of the barley pan-genome [13] and used for keyword searches of gene classes.

## 4. Discussion

Sourcing genetic resistance requires rigorous phenotypic testing of diverse accessions spanning different gene pools. Due to both historical and current changes in global political relations access to diverse accessions from some geographic regions such as Eastern Europe is challenging. In this study we used a GWAS-based mapping approach to mine a collection of diverse landraces and historical cultivars originally derived from Eastern Europe available through the Australian Grains Genebank for resistance to several barley infecting rust pathogen races from the US.

For consistency across the diverse pathogen races used we focused on phenotyping infection response at the seedling plant growth stage. The EEB accessions displayed a wide range of phenotypic responses to the seven different barley infecting cereal rust races spanning three distinct rust diseases of barley (stripe, crown and leaf rust). Most accessions (88%) were highly susceptible to the crown rust race used in this study. In the only other comparable study, Jin and Steffenson [34] assessed the response of 548 barley accessions from diverse geographic origins and found only 1.9% (10 accessions) that carried seedling resistance to crown rust. The higher frequency of resistance observed in the present study may be due to differences in virulence or presence of resistance in the respective isolates used or germplasm collections used for testing. Agrama et al. [9] mapped the first, and to our knowledge the only, crown rust resistance gene in barley to chromosome 3H, which was designated *Rpc1*. GWAS performed using the EEB accessions did not detect any signals on chromosome 3H, suggesting that *Rpc1* was absent, at very low frequency within the accessions or the isolate used in the study was virulent for *Rpc1*. However, four QTLs were identified (on 1HL, 2HS, 4HL and 5HL) that represent uncharacterised resistance alleles.

Although the EEBs were quantitatively assessed in separate experiments for their response to five pathogenically distinct *Psh* races, this was insufficient to efficiently postulate the presence of known *Rps* or tentatively designated barley stripe rust resistance genes within the 12 differential tester lines. Despite this, almost 90% of the EEBs were susceptible in response to PSH-72 relative to only 55% to the avirulent race PSH-48, suggesting the presence of previously identified race-specific resistances within this 35% of EEB lines. The 10% of accessions carrying resistance to PSH-72 represent previously undesignated or recently identified resistance sources. Furthermore, 16 of these lines carried either MR of HR infection types to all *Psh* races tested suggesting the presence of potentially multiple broadly effective resistance sources derived mainly from Russian, Czech and Greek descent. GWAS analysis revealed the presence of two broadly effective QTL on 5HL and 7HS responsible for resistance to races PSH-72 and PSH-100. The 7HS QTL was previously identified but barely significant in a previous study mapping seedling resistance to the same isolate (race PSH-72) and environmental conditions [35]. In our study the 7HS QTL was highly significant [−log10 (*p*-value) = 9.25 for PSH-72] which may reflect the frequency of the resistance in the population. Although not statistically significant based on the minor peak in the Manhattan plot, the 7HS QTL may also confer resistance to race PSH-54.

In contrast to resistance to the more avirulent PSH races, fewer EEB accessions were resistant to leaf rust (*P. hordei* race 17VA12C). Race 17VA12C is avirulent with respect to leaf rust resistance gene *Rph12*, suggesting that highly resistant EEB accessions (Infection type of 1) may carry *Rph12*. This was further supported by the GWAS results where a highly significant peak was identified on the distal region on the long arm of chromosome 5H where *Rph12* has previously assigned using morphological markers [36]. The two minor effect signals on 2HL and 4HL identified in response to *Ph* race 17VA12C may represent the previously cloned partial resistance QTL *Rphq2* [37] and an uncharacterised resistance QTL for 4H. The same peak marker for the 4HL QTL was also identified in response to barley crown rust, possibly suggesting the presence of a pleiotropic locus and conserved mechanism of resistance to biotrophic pathogens such as pathogenesis defence response genes. Further co-location of QTL across rust resistance traits was observed for the 5HL QTL effective to both leaf rust and stripe rust isolates used in this study. This was further supported by the highly significant Pearson’s coefficient between leaf and stripe rust traits the same EEB accessions were highly resistant to both leaf rust and stripe rust races carrying the 5HL QTL. Further examination of the physical interval harbouring the QTL identified the presence of a rich abundance of genes involved with resistance including receptor kinases, peroxidases etc. In both cases, multiple NLRs at the locus were identified which indicate that the pathogenicity effects could be controlled by different genes rather than a single gene as expected from major genes. Apart from the most renowned class of NBS-LRR immune receptors, other disease resistance gene classes have been described in the literature. We extracted genes in the analyzed interval from the Morex V3 genome annotation, where kinase-related genes and a further six disease-related genes were discovered in addition to the earlier predicted NBS-LRR genes (supplemental Table S2). This indicates the locus on 5H might resemble a complex of disease resistance candidate genes.

## 5. Conclusions

In conclusion, this study has determined the presence of several useful rust resistance alleles within a diverse collection of barley landraces and historic cultivars sourced from Eastern Europe. Further phenotyping of this collection is planned with necrotrophic pathogens both in the field and at the seedling stage to identify overlapping multi-pathogen QTLs.

## Figures and Tables

**Table 1:** Virulence (V)/Avirulence (A) spectra of five races of *Puccinia striiformis* f. sp. *hordei* on 12 barley stripe rust differential lines.

Races	Topper	Heils Franken	Emir	Astrix	Hiproly	Varunda	Adeb Binder	Trumpf	Mazurka	Bigo	I5	Bancroft
PSH-33	V	A	A	A	A	A	V	A	A	A	A	A
PSH-48	V	A	A	A	A	A	A	A	A	A	A	A
PSH-54	V	A	A	A	A	A	V	V	A	A	A	V
PSH-72	V	V	V	V	V	V	V	V	V	V	V	V
PSH-100	V	A	A	A	V	V	V	V	A	V	A	V

**Table 2:** Summary of the rust resistance QTL for five traits at the seedling stage identified in the Eastern European Barley accessions.

Trait	Marker	Chr	Pos (bp) *	Allele	QTL Interval	Flanking Markers	−log10(*p*-Value)	Effect on Trait
17VA12C	HvGBSv2-7726	2H	77796488	A	585594328	HvGBSv2-7726	7.63	−4.41
	HvGBSv2-9587	4H	558532757	C	558532757	HvGBSv2-9587	7.8	−2.64
	HvGBSv2-10463	5H	530696479	C	530058811–571321539	HvGBSv2-4369–HvGBSv2-4479	25.95	4.62
PSH-54	HvGBSv2-4385	5H	535795731	C	530058811–566858977	HvGBSv2-4369–HvGBSv2-10559	9.79	4.51
PSH-72	HvGBSv2-8315	3H	291597999	C	291597999	HvGBSv2-8315	6.26	1.93
	HvGBSv2-10469	5H	532621998	A	530696479–532621998	HvGBSv2-10463–HvGBSv2-10469	7.16	−2.24
	HvGBSv2-5420	7H	74751061	A	74751061	HvGBSv2-5420	9.25	−4.36
PSH-100	HvGBSv2-1099	2H	169669002	A	169669002	HvGBSv2-1099	6.38	7.33
	HvGBSv2-10469	5H	532621998	A	532621998	HvGBSv2-10469	6.88	−2.45
	HvGBSv2-5420	7H	74751061	A	74751061	HvGBSv2-5420	8.05	−4.36
91NE9305	HvGBSv2-520	1H	400297507	C	400297507	HvGBSv2-520	6.96	−2.02
	HvGBSv2-926	2H	47089419	C	47089419-55575144	HvGBSv2-926–HvGBSv2-928	5.9	2.35
	HvGBSv2-3492	4H	556987599	A	556987599	HvGBSv2-3492	5.84	−2.86
	HvGBSv2-10146	5H	348514954	C	387529130-393006859	HvGBSv2-10143–HvGBSv2-4058	6.28	2.72

* Position is based on the Morex v3 genome assembly described by Mascher et al. [12].

## Data Availability

All supporting data are available as supplementary tables and figures.

## Supplementary Materials

The following supporting information can be downloaded at: https://www.mdpi.com/article/10.3390/ijms241310860/s1.
