# Supplementary material for: Mining the Australian Grains Gene Bank for Rust Resistance in Barley
Source: Int J Mol Sci. 2023 Jun 29;24(13):10860. doi: 10.3390/ijms241310860 (PMC10342048; doi:10.3390/ijms241310860)
Supplement: Supplementary file 1 [file ijms-24-10860-s001.zip › Figure S1.pdf]

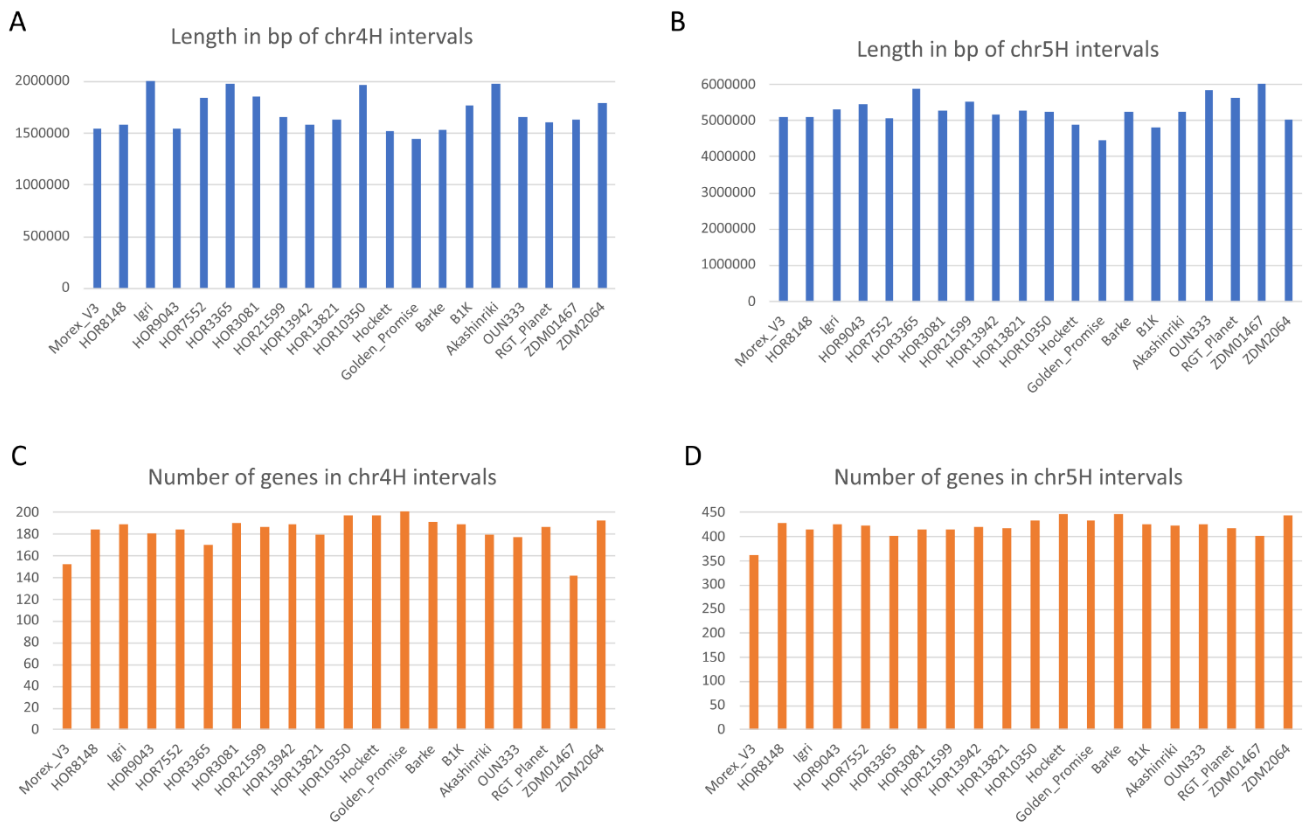

Figure S1. Physical length and gene content of candidate regions in the barley pan-genome. The upper two charts show the length of genome intervals on chromosome 4H (A) and 5H (B) corresponding to highly associated markers (Table 2, Figure 6) and their respective coordinates in the references of the barley pan-genome. The lower two charts show the number of predicted genes for chr4H (C) and chr5H (D).
